# Supplementary material for: Hierarchical modelling of functional brain networks in population and individuals from big fMRI data
Source: Neuroimage. 2021 Nov;243:118513. doi: 10.1016/j.neuroimage.2021.118513 (PMC8526871; doi:10.1016/j.neuroimage.2021.118513)
Supplement: Supplementary file 1 [file mmc1.pdf]

Appendices

A. Appendix to Introduction

In the Introduction, we focused on three key aspects that distinguish the existing single-subject and hierarchical models of functional brain modes, namely: direction of information flow between population and individuals, hierarchy on spatial topography and/or functional connectivity and scalability to big data. Here we present an extended set of factors, and compare sPROFUMO to some of the existing methods in more detail. The description of these factors is as follows:

- The direction of information flow between population and individuals: whether: a) the group-level estimations are used to regularise subject-specific estimations; or b) subject-specific models are combined to regularise the estimation of the group model or c) both. We refer to (a) and (b) as Unidirectional (Beckmann et al., 2009; Gordon et al., 2017; Kong et al., 2019; Mejia et al., 2019; Wang et al., 2015) and (c) as Bidirectional models (Abraham et al., 2013; Harrison et al., 2020, 2015; Manning et al., 2018; Shi and Guo, 2016; Varoquaux et al., 2011). Bidirectional models can also be iterative; where the model iterates between group- and/or subject-specific estimations until a convergence point is reached. s/PROFUMO is both bidirectional and iterative, where bidirectionality can be expected to accommodate more cross-subject heterogeneity, and model iterations can be expected to converge on more accurate subject-specific modes through optimisation.
- Explicit versus derivational mode estimations for population or individuals: whether mode estimations are explicitly done for each individual separately (Glasser et al., 2016; Harrison et al., 2020, 2015; Kong et al., 2019; Manning et al., 2018; Mejia et al., 2019; Shi and Guo, 2016; Wang et al., 2015) or subject modes are derived as a variant of the group (Abraham et al., 2013; Beckmann et al., 2009; Varoquaux et al., 2011). Furthermore, whether mode estimations are explicitly done for the group, or the group is an average of subjects. Explicit subject-specific modelling in s/PROFUMO can be expected to reduce biases towards population averages, and explicit s/PROFUMO group models can be expected to accommodate more cross-subject variance.
- Defining hierarchy over mode topography or functional connectivity: all hierarchical models attempt at finding a consensus group-level representation, such that subject modes will be comparable. SMAP hierarchy refers to when a model attempts at finding spatial mode layouts that are consistent across individuals (Glasser et al., 2016; Li et al., 2017; Manning et al., 2018; Mejia et al., 2019; Nickerson et al., 2017; Salehi et al., 2017; Shi and Guo, 2016). TNET hierarchy refers to when a model attempts to find between-mode temporal correlation matrices that are consistent across individuals (Chong et al., 2017). Defining hierarchy on both SMAPs and TNETs in s/PROFUMO (introduced in (Harrison et al., 2020)) can be expected to improve the reconstruction of sources of subject variability in spatial versus temporal properties of RSNs.

- Cross-session hierarchy: whether the model allows for an additional hierarchical mode estimation within subjects (i.e. across recording sessions), as incorporated in multi-session hierarchical Bayesian model (Kong et al., 2019). Cross-session hierarchy can be expected to more accurately assign within-subject variations and reduce the possibility of these variations being wrongly attributed to between-subject variations.
- Defining modes as distributed networks (Gordon et al., 2017; Harrison et al., 2015; Kong et al., 2019; Shi and Guo, 2016; Varoquaux et al., 2011; Wang et al., 2015) versus contiguous parcels (Abraham et al., 2013; Glasser et al., 2016; Manning et al., 2018). Both distributed and contiguous parcellations can be beneficial depending on study requirements. As elaborated in 4.3 and 4.8, low-dimensional sPROFUMO PFMs yield distributed functional modes while high-dimensional PFMs yield a mixture of distributed networks and contiguous parcels.
- Hard (Glasser et al., 2016; Gordon et al., 2017; Kong et al., 2019; Wang et al., 2015) versus soft (Beckmann et al., 2009; Harrison et al., 2020, 2015; Manning et al., 2018; Mejia et al., 2019; Shi and Guo, 2016) boundaries between modes. Both hard and soft parcellations can be beneficial, depending on study requirements. sPROFUMO yields soft parcellations.
- Scalability of algorithms to modern big data such as HCP (~1000 subjects) and UKB (~100,000 subjects). The latter is one of the main contributions of sPROFUMO, which has not been achieved previously using models that are bidirectional, iterative and incorporate explicit functional mode modelling in population and individuals.

B. Appendix to section 3.2.1: missing modes

In the main section 3.2.1 we discussed the issue of missing modes in PROFUMO framework and briefly outlined five key changes in the data and model that enabled us to largely resolve this issue in this study. Here we elaborate these five factors.

Reduced group-level missing modes using UKB and sPROFUMO

Here, when applying sPROFUMO to UKB, we found 46/50 and 92/100 non-empty modes at the group-level, which denotes an increase compared to our previous findings (Harrison et al., 2020). A few factors that can explain this observation are: firstly, the large number of subjects in UKB improved the signal-to-noise ratio of the group-level estimations, which can lead to a higher number of group-level modes being reliably reconstructed. Secondly, stochastic inference in sPROFUMO may have allowed for the VB optimisation to jump out of local minima, resulting in improved minima that seems to allow for higher degrees of subject variability in the group model. More specifically, in PROFUMO, a mode is typically returned as non-empty only if it is consensually estimable in a majority of the subjects (Harrison et al., 2020). sPROFUMO appeared to show more flexibility around such consensuses, such that modes can be estimated when present in a minority of the subjects too. Finally, in the previous papers we mostly found cortical modes, whereas here a subset of the new

modes that we discovered comprised subcortical and cerebellar RSNs. This can potentially be attributed to the type of data, in that here we utilised volumetric fMRI while in the previous papers we used CIFTI (UKB data has not yet been preprocessed into CIFTI form; this is planned for 2021). CIFTI data combines surface-based reconstruction of the cortical areas and volumetric reconstruction of the sub-cortical grey matter. As a result, there are known to be substantial differences in the smoothness of the data measured from cortical and subcortical regions, leading to inhomogeneities in the signal-to-noise ratio. Conversely, volumetric data yields more homogenous signal in different parts of the brain that can potentially help with recovering of the cortical and subcortical modes, alike.

Even though these three factors significantly decreased the number of the group-level missing modes, a number of subject-level missing modes persisted. We applied additional data curation strategies to reduce the remaining missing modes.

Additional data processing to reduce subject-specific missing modes

Firstly, we applied additional spatial smoothing using FSL tool *fslmaths* with Gaussian smoothing kernel sigma of 2.00, 2.05 and 2.15mm within cortex/white matter, cerebellar and subcortical masks, respectively. The slight difference in smoothing parameters were designed to account for the inherent smoothness differences in resting state signals in these regions. The final estimated smoothness, according to FSL's *smoothest*, was on average ~7.2mm FWHM across subjects in all the masks. We observed that this relatively small amount of additional smoothing makes a notable difference to the high dimensional sets of modes obtained from both sPROFUMO and ICA, especially for the estimation of single subject spatial maps and reducing missing modes.

As a part of subject-specific preprocessing, PROFUMO by default applies an SVD dimensionality reduction to subject data matrix $\mathbf{D}^{sr}$ in EQUATION 1 to give $\mathbf{X}^{sr} \in \mathbb{R}^{N_v \times N_p}$, where N_p is the number of top singular vectors. Subsequent matrix factorisations and mode inference are conducted in this dimension-reduced space. For low-dimensional mode decompositions (e.g. ~40 modes) this dimensionality reduction offers an efficient way of reducing the computational costs while largely preserving the estimation accuracies (provided the number of modes is small compared to the number of timepoints per subject). However, we found that even though the most prominent modes are consistent among the individuals, as we move up to higher dimensions, it becomes harder for the SVD to dissociate signal from noise components. Thus, the rank of the modes can arbitrarily change across individuals and more of the group-level modes will be lost in single subjects during SVD reduction. This results in those modes being returned as empty in individuals. In sPROFUMO, considering that only a small fraction of the population is kept in the memory for any given iteration, we are able to work with the full data matrix $\mathbf{D}^{sr}$. We found this change to greatly reduce the subject-specific missing modes at high dimensions; since, even if a mode does not explain much of the data variance, it can still be recovered in single subjects.

C. Appendix to simulation set 2 (section 4.2)

In the main section 4.2 we presented three simulation scenarios associated with challenges of high-dimensional mode decomposition from resting state fMRI data. FIGURE A 1 shows examples of simulated ground truth modes and estimated sPROFUMO modes. In the main text, we compared sPROFUMO results to that of ICA and ICA-

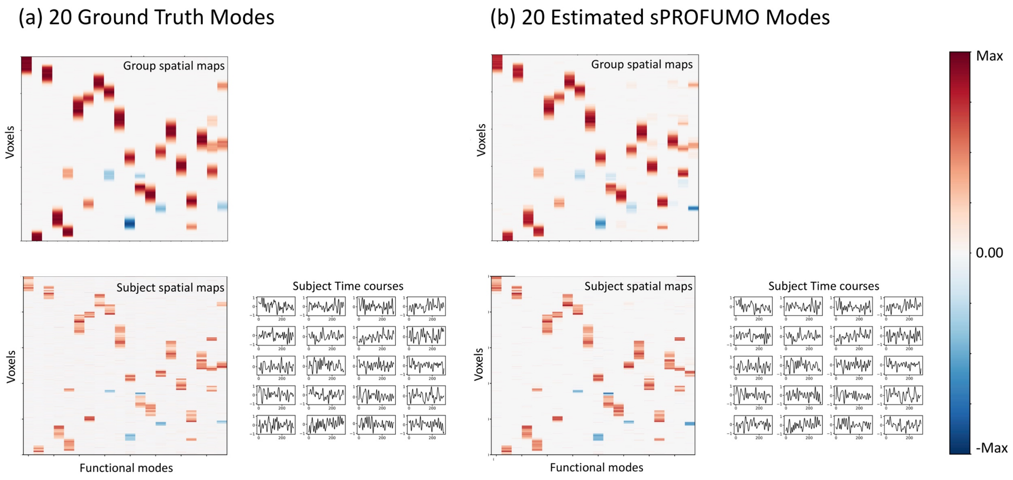

Figure A 1 Examples of a) simulated and b) estimated networks in the presence of spatial overlap among the modes and spatial misalignment across subjects. Top: group-level spatial maps. Bottom: subject-level spatial maps and time courses for an example simulated subject.

Dual Regression (ICA-DR). Here in FIGURE A 2 we show an extended version which also includes classic PROFUMO. Based on these results, in the presence of different degrees of cross-subject variability in the modes, spatial overlaps between the modes and smaller mode sizes, sPROFUMO and PROFUMO show similar performances and they both outperform ICA and ICA-DR. The difference is particularly notable for single subject estimation of the spatial mode topographies where s/PROFUMO tend to yield accuracies that are up to ~100% superior to that of ICA/-DR.

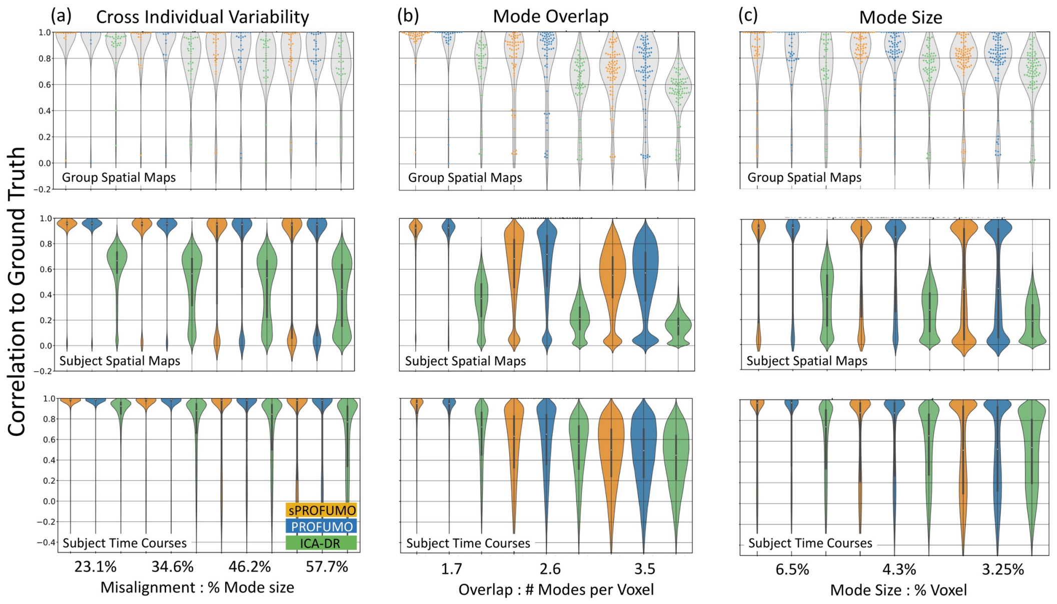

Figure A 2 Results of simulation set 2 (section 4.2) when including PROFUMO.

Modes with low accuracies

A closer look at the violin plots in FIGURE A 2 (and FIG. 4) reveals a puzzling behaviour: estimation accuracies of the spatial maps sometimes depict a bimodal distribution, where a subset of the modes show accuracies near zero while a majority of the modes are estimated very accurately; e.g. see the distribution where the misalignment is 57.7% in FIG. 4A.

A subset of these low accuracy modes can be attributed to when a ground truth mode is missing from the estimations. This is likely to happen at both group- and subject-level, e.g. when a mode is excessively small and/or noisy, such that it cannot be estimated as one of the top modes, and instead is included within the residuals. An additional factor that can give rise to subject-specific missing modes in these simulation scenarios, and consistent with the pattern of results that we observe, is that they occur when a group map is not representative of the entire population. For example, if the amount of misalignment in a subset of subjects is so large that a specific mode has minimal overlap with the group, then it will be difficult for both sPROFUMO/PROFUMO and ICA-DR to estimate those subjects accurately. Arguably, this scenario will also be challenging for other hierarchical algorithms that aim to find one single consensus group model, considering that a key assumption of these models will be violated.

The low accuracy modes are also likely to be false positives; i.e. where a mode that does not exist in the ground truth is detected by the matrix factorisation model. Such false positives might occur when the assumptions of matrix factorisation model (e.g. spatial independence in spatial ICA) are largely violated in the ground truth, or a noise-driven component being erroneously detected as a functional mode.

D. Appendix to summary of UK Biobank results (section 4.3)

In the main section 4.3 we showed the results of applying sPROFUMO to resting state fMRI data of 4999 subjects from UK Biobank and characterising 150 functional modes. Here we present supplementary figures to that section. FIGURE A 3 shows model convergence based on full population Free Energy (output from stochastic Variational Bayesian optimisation process) as well as group-level spatial maps and partial temporal NetMats. FIGURE A 4 is linked to the main section 4.3.1 and shows how explicit decomposition of modes’ spatial and temporal properties into signal and noise components help finding a clean estimation of the subject-specific modes that is minimally contaminated by noise. This also shows how high-SNR large-scale RSNs, low-SNR large-scale RSNs and parcel-like RSNs differ with respect to the noise levels.

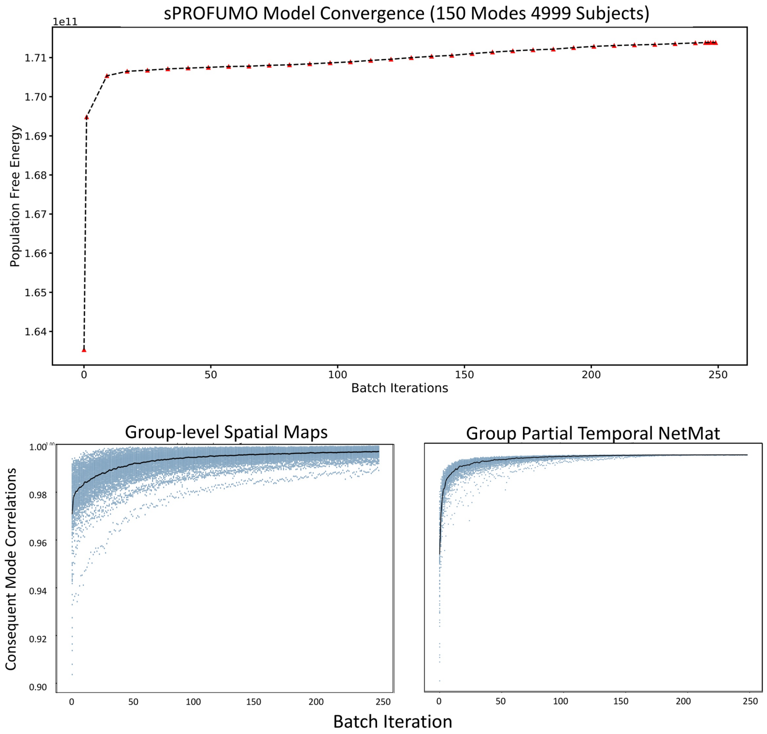

Figure A 3 sPROFUMO model convergence on UKB data. Top: Free Energy convergence of the full model; bottom: correlation of group spatial maps and group partial temporal NetMats in each batch iteration (i), with their immediately preceding iteration (i-1) until convergence.

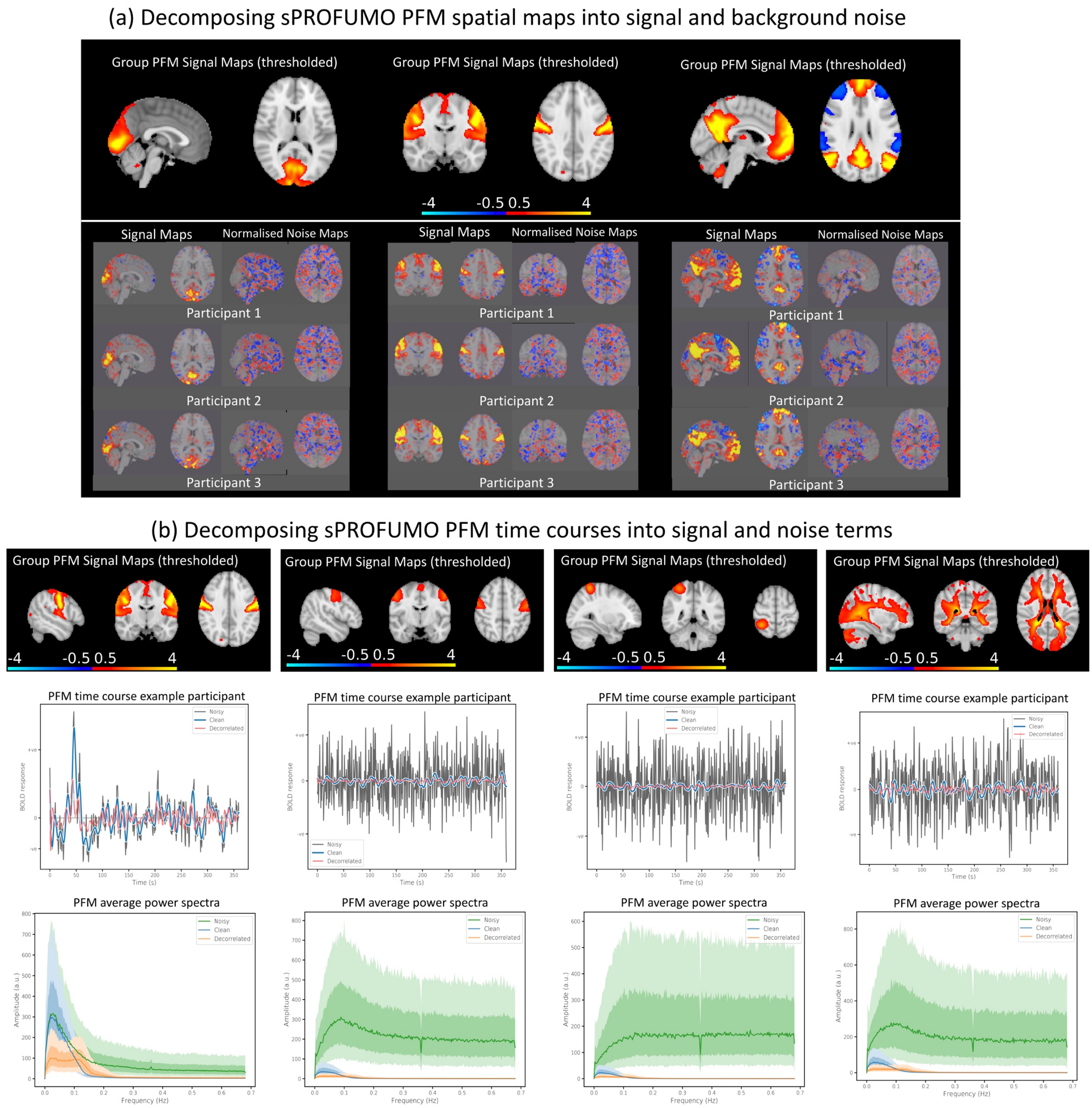

Figure A 4 Signal and noise terms in spatial and temporal aspects of sPROFUMO PFMs: a) group-level spatial signal maps (top) and subject-level spatial signal and noise maps for three random participants. Signal maps in subjects denote the spatial mode topography and noise maps denote the background; b) examples of one PFM per each of the four categories from FIG. 5: 1) high-SNR distributed (first left), 2) low-SNR distributed (second left) and 3) low-SNR parcel-like (third left) sPROFUMO RSNs in parieto-central cortices as well as 4) a mode with physiological/acquisition origin (right). Note that three time courses/power spectra are shown in each plot which include: clean time course of the signal component only, decorrelated time course of the signal component after de-convolving with an HRF response function, and noisy time course from combined signal and noise components.

E. Appendix to stability of sPROFUMO PFMs (section 4.3.2)

This section provides a detailed description of the results that were presented in 4.3.2. We evaluated how reliably sPROFUMO modes were estimated across subjects and model runs based on two set of consistency metrics:

Firstly, we tested between-run stability of the sPROFUMO's results, by re-running the model on the same subjects and measuring correlations between the outputs of the two runs. In stochastic inference, due to the randomisation over the local variables, different runs of a model are prone to yielding different results, even if the inference is conducted based on an identical set of parameters and on the same data. Despite this property, our aim is that the final sPROFUMO output remains stable across multiple runs. To test the stability, we initialised the model based on the same set of initial maps and priors, and re-inferred subject and group PFMs. Results are shown in FIGURE A 5A, where we found average consistency of 0.98, 0.98 and 0.94 for the group spatial maps, spatial and temporal NetMats, respectively. For subject-specific sPROFUMO PFMs these values were: 0.90, 0.94 and 0.77, with 0.85 consistency for the amplitudes.

Secondly, we compared sPROFUMO modes obtained from 1500 subjects with results from 4999 subjects. The 1500 subjects were selected to be a subset of the original 4999 subjects, in order to allow us to test for the replicability of PFMs at both group- and subject-level. While we expect larger population sizes to unravel richer patterns of group and subject variability, this comparison will allow us to test if applying sPROFUMO to smaller populations will produce comparable PFMs with those obtained from larger populations. Results are shown in FIGURE A 5B, where we found average replicability of 0.96, 0.92 and 0.86 for the group spatial maps, spatial and temporal NetMats, respectively. For subject-specific PFMs, these values were: 0.84, 0.72 and 0.62, with 0.89 consistency for the amplitudes. The lower replicability of temporal NetMats can be traced back to the structure of off-diagonal elements in FIG. 5B-RIGHT, where a large number of partial correlations are small and near-zero. These off-diagonal elements can be expected to be affected by noise, thus yielding lower subject-level reproducibility in smaller population.

Thirdly, we measured cross-individual robustness of the results based on subject-to-group (S2G) and subject-to-subject (S2S) consistencies. In the absence of a ground truth in the real data, higher S2G and S2S consistencies are often used as metrics of performance and robustness in single subject modelling (Gordon et al., 2020; Guntupalli et al., 2018). This is due to the fact that, firstly, we expect the biologically meaningful RSNs to exhibit similar key features across individuals (e.g. right-hand motor network should localise to left motor cortex), and we expect the group model to capture the key elements shared across the population. Secondly, the model should ideally be able to remove the effect of measurement noise from single subject mode estimations (e.g. see FIGURE A 4) which in turn is expected to increase S2G and S2S values. We measured S2G consistencies by finding correlation coefficients of the corresponding mode elements (e.g. spatial maps) between a subject and the group. Similarly, for S2S consistencies, we correlated each subject's mode elements with all other subjects. Pooling the results in Raincloud plots (FIGURE A 5C) revealed that S2G consistencies were generally ~10% higher than S2S consistencies. We further found the most-to-least consistent mode elements to be: spatial NetMats

(S2S: 0.79±0.017, S2G: 0.89±0.019), partial temporal NetMats (S2S: 0.70±0.085, S2G: 0.83±0.10), spatial maps (S2S: 0.55±0.024, S2G: 0.73±0.033) and mode amplitudes (S2S: 0.31±0.055, S2G: 0.56±0.093). It is worth noting that while S2G and S2S consistencies yield useful metrics of results stability, they do not inform us of the relationship between estimated and ground truth subject-specific variability in spatial and/or temporal domains. Therefore, we complement results from this section with additional metrics from simulations (4.2) and prediction power for cognitive tests (4.6) to illustrate model's ability to accurately and meaningfully capture cross-subject variations.

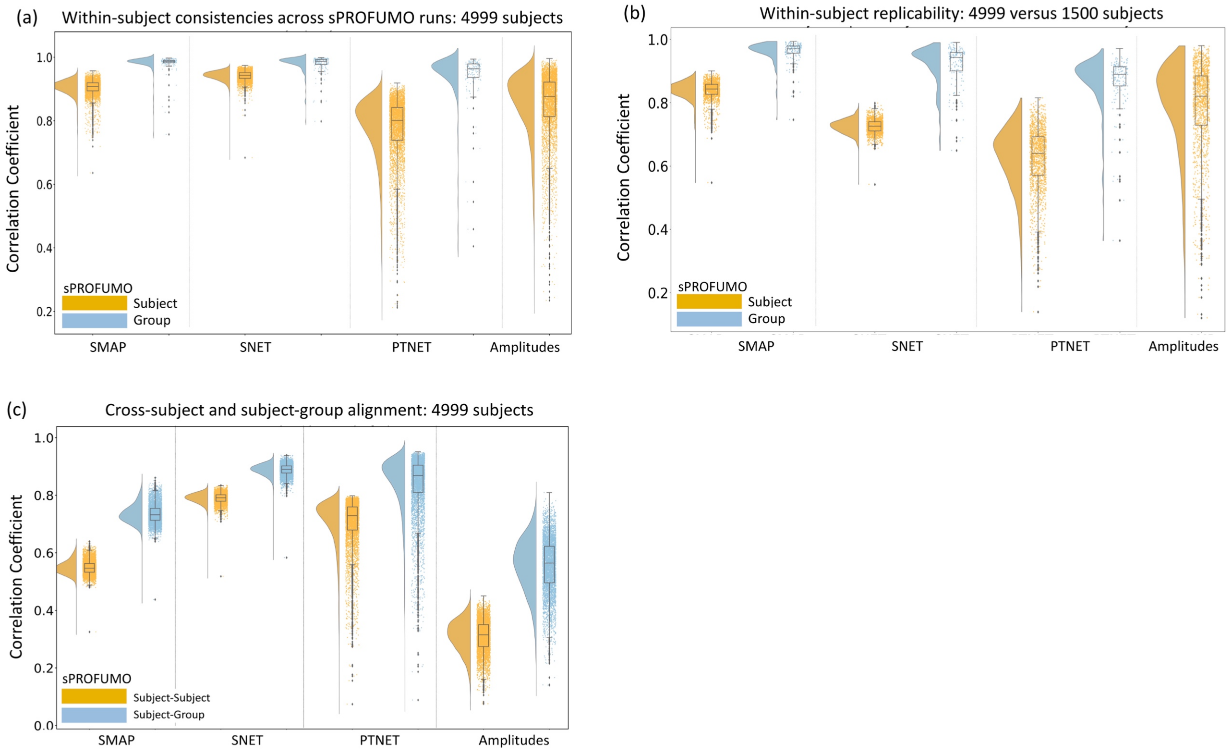

Figure A 5 Stability of sPROFUMO modes: a) consistency of group- and subject-level PFMs across two sPROFUMO runs; b) consistency of group- and subject-level PFMs when running sPROFUMO on two population sizes, 1500 and 4999 subjects; c) subject-to-group and subject-to-subject consistency of PFMs within a single model run. Consistencies are measured based on different model elements including spatial maps (SMAP), spatial and partial temporal NetMats (SNET, PTNET), and Amplitudes. Note that we do not include a Raincloud plot (Allen et al., 2019) for the group-level amplitudes in panel (a) because each mode has one group-average amplitude thus yielding 150 values per model run. The correlation between these amplitudes is therefore just a single number.

F. Appendix to comparison of sPROFUMO and ICA-DR (section 4.5)

In the main section 4.5 we showed how high-dimensional sPROFUMO decomposition of 150 modes compared to that of ICA and ICA-DR. Figure A 6 is supplement to Fig. 7 and shows how distribution of subject spatial maps in different brain voxels differs between sPROFUMO spatial signal element and ICA-DR.

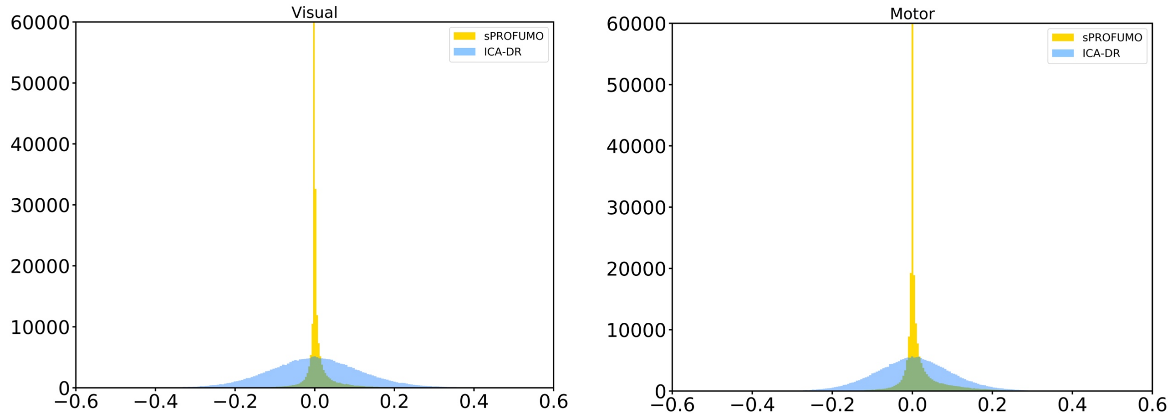

Figure A 6 Supplement to Fig. 7: histograms of sPROFUMO subject spatial maps (signal element) and ICA-DR spatial maps across voxels for an example participant and two example modes. Left: visual modes in Fig. 7, Right: motor modes in Fig. 7.

G. Appendix to prediction results (section 4.6)

In the main section 4.6 we showed results of using 150 sPROFUMO modes from resting state fMRI from 4999 UK Biobank subjects to predict cognitive outcome.

FIGURE A 7 shows that prediction results are maintained regardless of the choice of deconfounding. FIGURE A 8 shows sPROFUMO's multi-mode multi-element prediction accuracies for every cognitive test separately (refer to the main text for additional explanations). TABLE A 1 shows the names of cognitive tests included in predictions and full details of each test are available in UKB website: https://biobank.ctsu.ox.ac.uk/crystal/label.cgi?id=100026. FIGURE A 9 compares sPROFUMO's multi-mode prediction accuracies to PROFUMO for 1500 subjects and 150-mode decomposition.

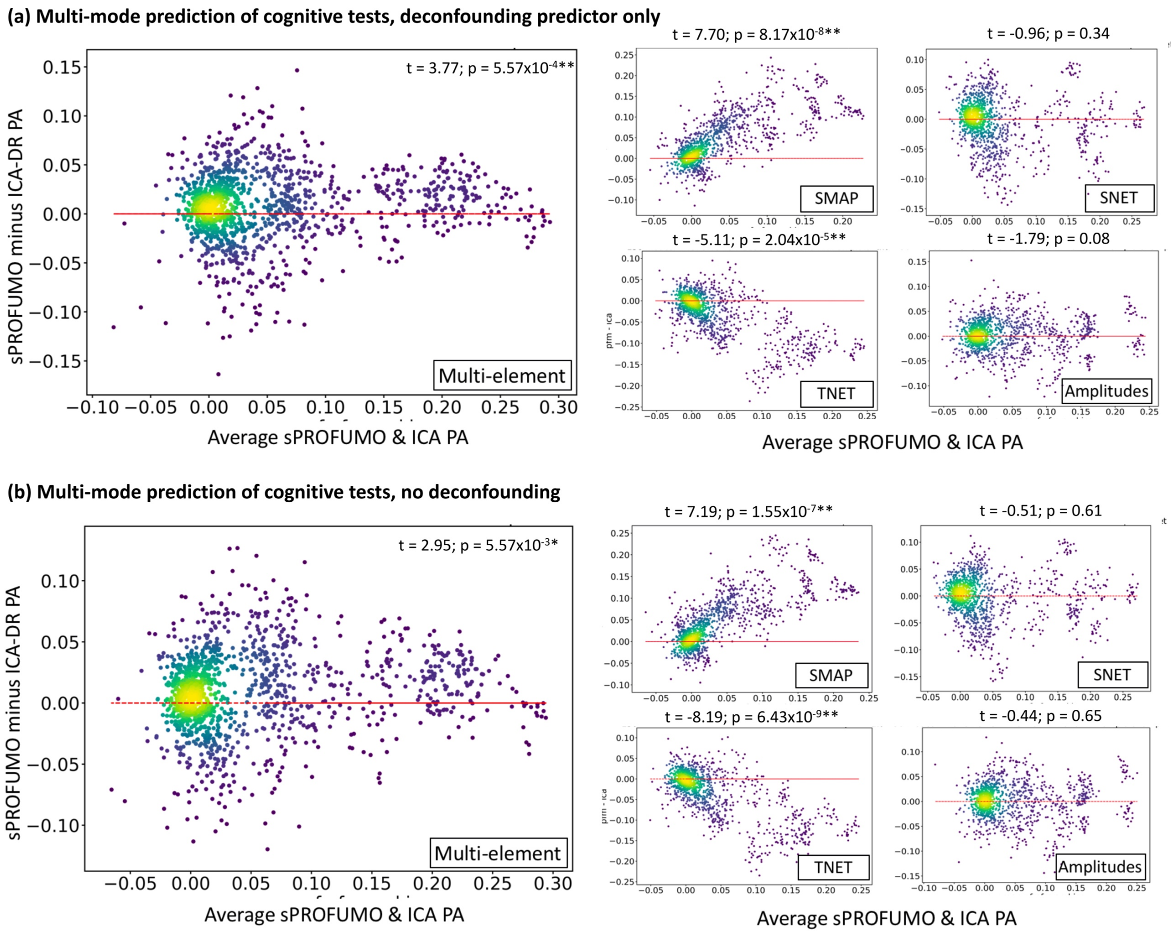

Figure A 7 Multi-element prediction results based on different deconfounding strategies: a) deconfounding predictor variable only; b) no deconfounding. Comparison to FIG. 8A shows that results and conclusions are maintained regardless of the choice of deconfounding.

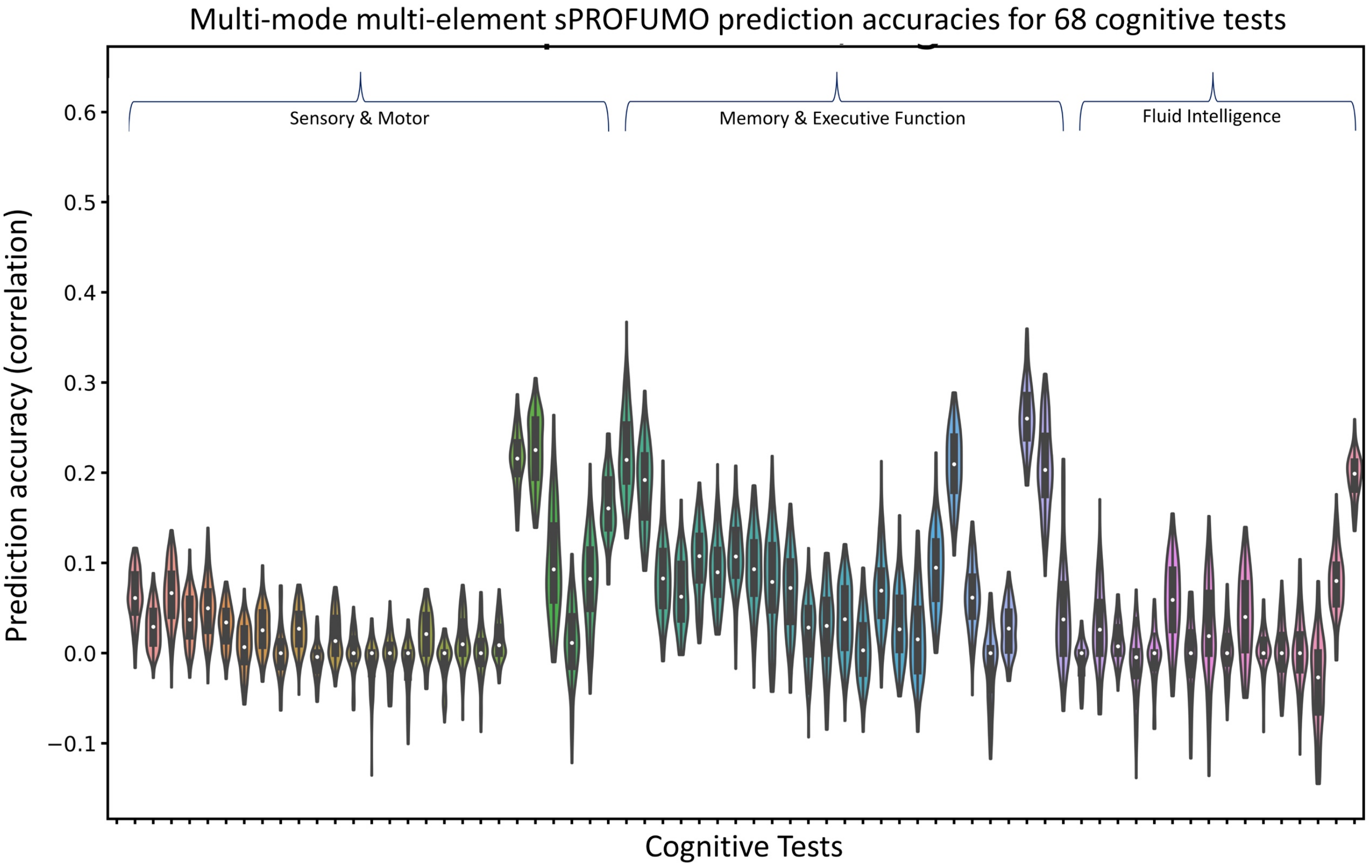

Figure A 8 Accuracies of sPROFUMO's multi-mode multi-element predictions for each of 68 cognitive tests. 5-fold cross-validations are repeated 20 times to obtain distributions for violin plots. The model is generally best at predicting higher cognitive functions such as memory and executive function followed by sensorimotor function and fluid intelligence scores. Best-predicted tests include: Mean time to correctly identify matches (0.0), Mean time to correctly identify matches (2.0), Duration to complete alphanumeric path (trail #2) (2.0), Duration to complete numeric path (trail #1) (0.0), Duration to complete alphanumeric path (trail #2) (0.0), Time elapsed [in numeric memory test] (2.0), Time elapsed [in numeric memory test] (2.1), Maximum digits remembered correctly (2.0), Number of puzzles correctly solved (2.0), Number of symbol digit matches made correctly (0.0), Number of symbol digit matches made correctly (2.0), Fluid intelligence score (2.0).

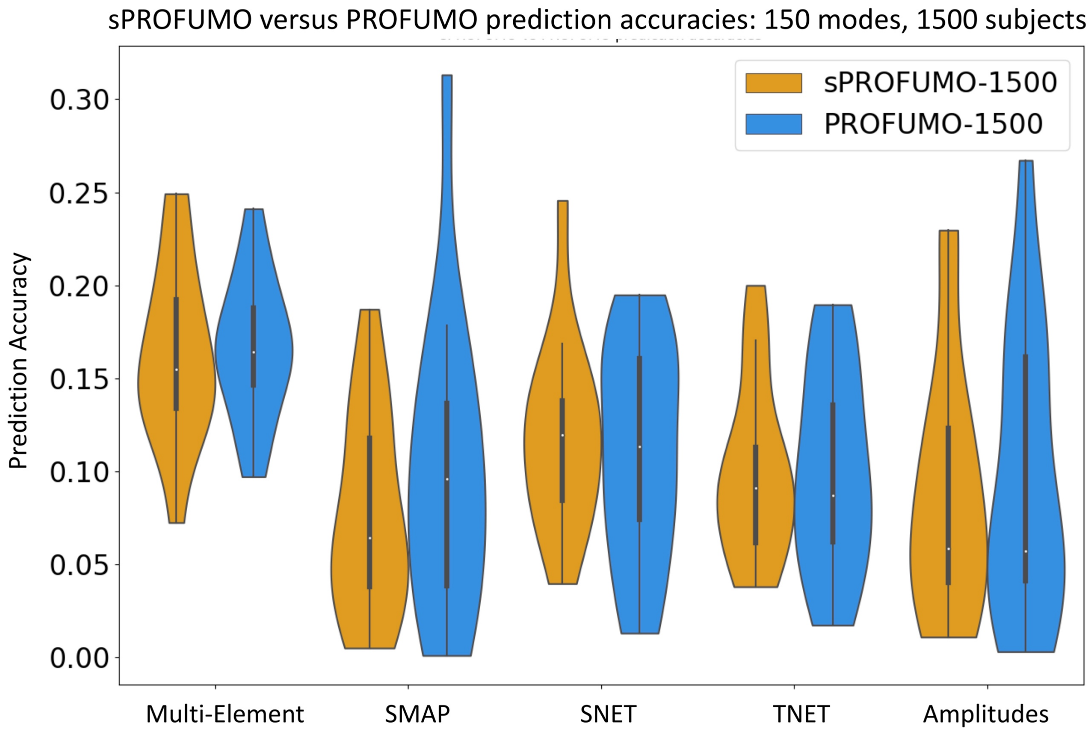

Figure A 9 Comparing prediction accuracies of PROFUMO and sPROFUMO: multi-mode predictions used to compare model performances based on 150-mode decompositions of 1500 subjects. Pairwise comparisons based on weighted paired t-tests revealed no significant differences between the two models (all p-values > 0.3). SMAP: Spatial Maps; SNET: Spatial NetMats, TNET: Temporal NetMats.

Table A 1 68 cognitive tests used for prediction.

Cognitive tests		
1- No. incorrect matches (0.1)	31- Digits correctly (2.1)	60- Prospective memory (2.0)
2- No. incorrect matches (0.2)	32- Digits correctly (2.2)	61- Mn time identify matches (0.0)
3- No. incorrect matches (2.1)	33- Digits correctly (2.3)	62- Mn time identify matches (2.0)
4- No. incorrect matches (2.2)	34- Digits correctly (2.4)	63- No. incorrect matches (0.0)
5- No. incorrect matches (2.3)	35- Digits correctly (2.5)	64- Dur num. path (trl #1) (0.0)
6- No. times snap-button (0.0)	36- Digits correctly (2.6)	65- Dur alphanum path (trl #2) (0.0)
7- No. times snap-button (0.1)	37- Digits correctly (2.7)	66- No. symbol digit matches (0.0)
8- No. times snap-button (0.2)	38- Max digits correctly (2.0)	67- No. symbol digit matches (2.0)
9- No. times snap-button (0.3)	39- FI1 : numeric add. (0.0)	
10- No. times snap-button (0.4)	40- FI1 : numeric add. (2.0)	
11- No. times snap-button (0.10)	41- FI3 : word interpol. (0.0)	
12- No. times snap-button (0.11)	42- FI3 : word interpol. (2.0)	
13- No. times snap-button (2.0)	43- FI4 : posit. arithmetic (0.0)	
14- No. times snap-button (2.1)	44- FI4 : posit. arithmetic (2.0)	
15- No. times snap-button (2.2)	44- FI5 : fam. rel. calc. (0.0)	
16- No. times snap-button (2.3)	45- FI5 : fam. rel. calc. (2.0)	
17- No. times snap-button (2.4)	46- FI6 : condit. arith. (0.0)	
18- No. times snap-button (2.5)	47- FI6 : condit. arith. (2.0)	
19- No. times snap-button (2.7)	48- FI7 : synonym (0.0)	
20- No. times snap-button (2.10)	49- FI7 : synonym (2.0)	
21- No. times snap-button (2.11)	50- FI8 : chained arithmetic (2.0)	
22- Time elapsed (2.0)	51- FI9 : concept interpol. (2.0)	
23- Time elapsed (2.1)	52- FI10 : arith. seq. recog. (2.0)	
24- Time elapsed (2.2)	53- Dur. num. path (trl #1) (2.0)	
25- Time elapsed (2.3)	54- Total err num. path (trl #1) (2.0)	
26- Time elapsed (2.4)	55- Dur alphanum. path (trl #2) (2.0)	
27- Time elapsed (2.5)	56- No. puzzles correct (2.0)	
28- Time elapsed (2.6)	57- Fluid intelligence (0.0)	
29- Time elapsed (2.7)	58- Fluid intelligence (2.0)	
30- Digits correctly (2.0)	59- Prospective memory (0.0)	

H. Appendix to effect of mode dimensionality (section 4.8)

In section 4.8 we showed the effect of mode dimensionality on sPROFUMO results, comparing 150 modes with 100 and 200. FIGURE A 10 shows the convergence rates of the group spatial maps and partial temporal NetMats based on correlations between the group model obtained in each model iteration with the immediately preceding group model. FIGURE A 11 compares prediction accuracies of the primary and secondary PFMs and illustrates examples of well-performing secondary PFMs.

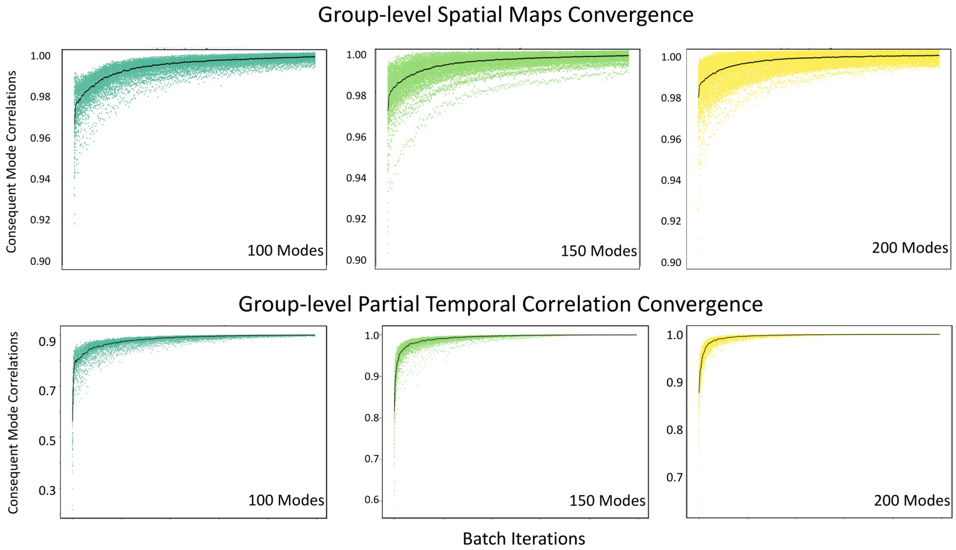

Figure A 10 Convergence rates of different dimensions of sPROFUMO modes at rest. correlation of group spatial maps and group partial temporal NetMats in each batch iteration (i), with their immediately preceding iteration (i-1) until convergence.

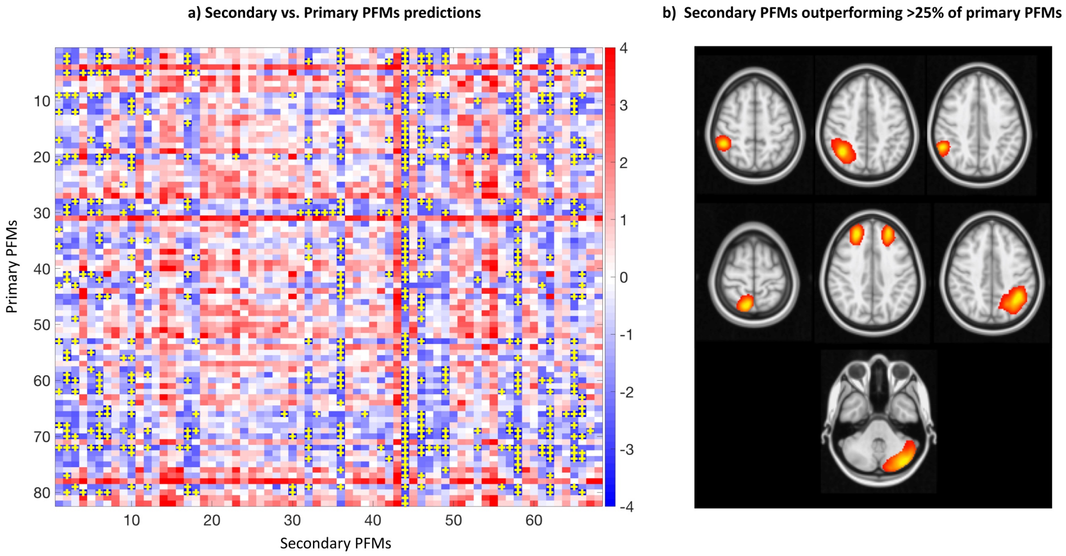

Figure A 11 Comparing secondary PFMs to primary PFMs for prediction of cognitive tests. As outlined in 4.8, we categorised PFMs from 150-mode decomposition into two groups: 82 primary PFMs and 68 secondary PFMs. The latter are fine-grained modes that are found as we move to higher dimensions. a) t-values from weighted paired t-test comparison of multi-element prediction accuracies of every secondary PFM to all the primary PFMs. Blue shows primary<secondary, red vice versa. Locations marked with + denote secondary PFMs that are significantly better than primary PFMs, after FDR correction for multiple comparisons (across 68x82 tests). b) based on results in (a), seven secondary RSNs were found to outperform >25% of the primary RSNs for prediction of cognitive tests.

Supplementary References

Allen, M., Poggiali, D., Whitaker, K., Marshall, T.R., Kievit, R.A., 2019. Raincloud plots: a multi-platform tool for robust data visualization. Wellcome Open Res. 4. https://doi.org/10.12688/wellcomeopenres.15191.1

Salehi, M., Karbasi, A., Scheinost, D., Constable, R.T., 2017. A submodular approach to create individualized parcellations of the human brain, in: Lecture Notes in Computer Science (Including Subseries Lecture Notes in Artificial Intelligence and Lecture Notes in Bioinformatics). https://doi.org/10.1007/978-3-319-66182-7_55

Wang, D., Buckner, R.L., Fox, M.D., Holt, D.J., Holmes, A.J., Stoecklein, S., Langs, G., Pan, R., Qian, T., Li, K., Baker, J.T., Stufflebeam, S.M., Wang, K., Wang, X., Hong, B., Liu, H., 2015. Parcellating cortical functional networks in individuals. Nat. Neurosci. 18, 1853–1860. https://doi.org/10.1038/nn.4164
